# Workplace Support for Mental Health Workers Who Are Parents: A Feasibility Study

**DOI:** 10.3389/fpsyg.2022.854065

**Published:** 2022-06-23

**Authors:** Abby Dunn, Clare Dixon, Abi Thomson, Samantha Cartwright-Hatton

**Affiliations:** ^1^Department of Psychology, University of Sussex, Falmer, United Kingdom; ^2^Sussex Partnership NHS Foundation Trust, Hove, United Kingdom

**Keywords:** parenting, mental health workforce, burnout, stress, staff development, work – family conflict

## Abstract

**Background:**

Mental health workers are subject to high levels of occupational stress which is associated with poorer health and wellbeing and impaired patient outcomes. For individuals operating in high stress environments, reducing challenge at home, in particular around parenting, has been found to generalize into improvements in the professional domain. The present study sought to investigate the effectiveness and feasibility of brief targeted workplace intervention to support workers in terms of their parental role.

**Design/Methodology:**

An uncontrolled evaluation of a series of three-session parenting-focused courses delivered to employees of a large Mental Health Trust. A pre-post-follow-up design was used to investigate effects on outcomes including parenting practice and experience, wellbeing, stress, and occupational self-efficacy. Intervention feasibility and acceptably was also evaluated.

**Findings:**

Data from 15 participants who completed measures pre-post indicates the courses were associated with improved parenting practice and experience at a *p* < 0.005 level. Improvements were reported at 6-month follow up. Participant satisfaction and course acceptability was highly rated by 100% of participants.

## Introduction

Mental health workers are amongst the most vulnerable to burnout of any health care professionals (Johnson et al., 2018). Rates of workplace stress amongst the mental health workforce have increased by 10% in a decade, with 41.2% of workers stating that stress led them to feel unwell in 2019. A 2018 meta-analysis identified a similar proportion of mental health workers to be operating at the level of emotional exhaustion, which is the core component of burnout (O’Connor et al., 2018). Unsurprisingly, mental health staff have higher levels of sickness-related absence than other medical sectors (Johnson et al., 2018). These figures represent the pre-COVID mental health workforce, with the most recent NHS Staff Survey indicating an 8% increase in workers experiencing elevated stress, and this is likely to be a conservative representation of the impact of the pandemic (NHS England, 2021a).

The challenges faced by individuals working in the mental health sector are complex, encompassing organizational stressors such as staffing shortages and workload, as well those which relate to the emotional burden carried by workers. This latter category includes violence toward staff, as well as the experience of involuntary detentions, suicidality and self-harm in patients (Johnson et al., 2018). While these experiences are not universal to all members of the mental health workforce, the majority are engaged directly or indirectly with individuals in distress and do so within an underfunded and oversubscribed service context. This has an impact in terms of staff wellbeing, the care they offer and leads to depletion of the workforce *via* absence and turnover, which in turn further contributes to fragility of an understaffed and overloaded system (Paris and Hoge, 2010; Johnson et al., 2012; O’Connor et al., 2018).

For individuals who experience high levels of work-related stress, an additional risk factor relates to the intersection of their professional and family life. Work-family conflict (WFC) describes the tension when a professional’s work and domestic roles are in conflict, which leads to impaired ability to perform in one of both domains (Greenhaus and Beutell, 1985). WFC comprises two bidirectional components, work interference with family life and family interference with work (Netemeyer et al., 1996). For example, the spill-over of employment-related stress can lead to impaired parental communication and engagement which may contribute to an escalation in child behavioral difficulties. This, in turn, increases stress at home, which can then lead to poorer engagement and function in the professional domain. WFC has also been found to mediate between job demands and psychological outcomes such as depression and burnout. In a study of Greek physicians, WFC was found to explain the relationship between work-related burden and emotional exhaustion. This was replicated in a survey of Japanese mental health nurses, where WFC was found to have a mediating effect on the relationship between job-related stress and burnout. Given the role WFC plays in burnout, this suggests workers within the mental health workforce are multiply vulnerable: operating in a stressed and stressful system which is likely to contribute to elevated levels of WFC, which in turn leads to elevated risk of burnout. It is unsurprising that mental health workers aged under 45 are most vulnerable to emotional strain, a core component of burnout (Johnson et al., 2012), given that these are the workforce members most likely to be parents of school-age children and as such more likely to experience conflict between their home and work domains.

In this representation of a multi-stressed workforce, it is important to recognize that while relatively common, burnout and chronic stress are not universal. Furthermore, just as the emotional burden of work can be exacerbated by the challenges of home, sources of resilience in one domain can operate as protective in another. Similarly, a reduction in stress within one domain can result in a reduction in the other. Many of the role and sector-based stressors experienced by the mental health workforce are unlikely to change in the foreseeable future; indeed the burden may intensify as the full impact of the pandemic is revealed (Byrne et al., 2021). Given the bidirectional transmission of stress from work to home, one method to improve the experience of the workforce is by targeting the domestic domain, in particular parenting role and experience.

The administration of health and wellbeing interventions in the workplace has risen as employers seek to prevent costs associated with sickness and absenteeism (Proper and Van Oostrom, 2019). While initiatives have been deployed to engage with varied outcomes including obesity, smoking cessation and mental health, interventions focused on the specific challenges faced by working parents are less common. Those that actively target parents fall into two broad categories: the first address WFC by engaging with the parent with regard to their working role, for example supporting workers to manage time better and reduce “presenteeism” (McHale et al., 2016). The second form of intervention is orientated to the parenting role of the worker. For example, delivering a tailored version of a widely-used behaviorally based parenting course to Australian teachers who were parents led to improved outcomes including in parental self-efficacy, parenting satisfaction, and a reduction in dysfunctional parenting and reduced workplace stress (Haslam et al., 2013). The acceptability of interventions designed to help parents manage the competing demands of work and home is high, with 80% of working parents stating they would attend a workplace parenting intervention (Sanders et al., 2011). A separate survey of working mothers identified a specific wish for brief interventions which incorporated tools and techniques orientated to parenting challenges such as behavior management (Haslam et al., 2015).

Within mental health services, there is scope to engage with workers in terms of their parental role with the potential for positive effects in terms of stress within the professional domain. Despite the opportunities afforded by this form of engagement, there is no evidence of a workplace intervention designed to do so in the United Kingdom mental health service context. The current study reports on the feasibility of a brief targeted intervention to support parents working in one United Kingdom mental health service. This took the form of a series three session courses run across a large mental health trust in the South of England designed to achieve the following outcomes: improvement in the parenting practice and experience of participants, improvements in wellbeing, stress, and occupational self-efficacy. Intervention feasibility and acceptably was also evaluated.

## Materials and Methods

### Ethics

The authors assert that all procedures contributing to this work comply with the ethical standards of the relevant national and institutional committees on human experimentation and with the Helsinki Declaration of 1975, as revised in 2008. All procedures involving human subjects/patients were approved by HRA and Health and Care Research Wales.

### Recruitment

Sussex Partnership NHS Foundation Trust (SPFT) is a large Mental Health Trust operating across more than 100 sites in East Sussex, West Sussex, Brighton and Hove and Hampshire. It employs more than 4,500 staff organized into 430 teams. The CEO of the Trust was instrumental in the initiation of the program and had authorized staff to take part in the sessions during their core working hours. The recruitment process was designed to encourage staff members who were parents to feel able to take time out from work to participate. Recruitment strategies included visiting leadership meetings to promote the project and gain buy-in with team leads, communication to staff direct from the CEO of the Trust and inclusion in the weekly wellbeing newsletter sent to all staff by the HR Service. These activities were accompanied by localized promotional posters and advertisements on the Trust intranet.

Potential participants were able to sign-up directly for a course using an online ticketing system or could email the project team. Participants could apply to join any course and if their chosen course was full, they were invited to join a waiting list.

### Participants

A broad inclusion criterion was used to maximize engagement. Participants were eligible if they were a current employee of the Trust and were a parent or carer to a child aged between 2 and 11 who resided with them for at least part of the week. A parent had to have agreement from their manager that they could take time away from their usual work in order to participate.

The course (comprising three sessions) was scheduled to run with four separate groups of participants. The final two courses were terminated prematurely in response to the COVID-19 epidemic. Only demographic data from the two courses which ran to completion is reported.

Of the two completed courses, a total of 17 participants attended the first session (Group 1 = 8, Group 2 = 9). Most participants classified themselves as female (*n* = 76.5%) with an average age of 40 years (*SD* = 7.2, range 30–57 years). Most participants had more than one child (range 1–4) with ages ranging from 1 to 12 years.

Most participants identified as being from a clinically focused team (*n* = 12, 70.6%) and holding a clinical role (*n* = 11, 64.7%). The average duration of service at the Trust was 7 years and 10 months (*SD* = 73.0, range 2 months to 19 years). Demographic and professional characteristics disaggregated by group are reported in full in Table 1.

**TABLE 1:** Participant demographic data displayed by group.

		Group 1	Group 2	Total
		
Participants		8	9	17
		
		N (%)	N (%)	N (% of sample)
**Gender identity**				
	Female	5 (62.5)	8 (88.8)	13 (76.5)
	Male	3 (37.5)	1 (11.1)	4 (23.5)
**Ethnicity**				
	White British	8 (100)	8 (88.9)	16 (94.1)
	White Other	–	1 (11.1)	1 (5.9)
**Disability**				
	No	8 (100)	8 (88.9)	16 (94.1)
	Yes – a little	–	1 (11.1)	1 (5.9)
**Team**				
	CAMHS	2 (25.0)	2 (22.2)	4 (23.5)
	Administration	1 (12.5)	1 (11.1)	2 (11.8)
	Assessment and Treatment		3 (33.3)	3 (17.6)
	Community	3 (37.5)	–	3 (17.6)
	Early Intervention	–	1 (11.1)	1 (5.9)
	Estates and Facilities	1 (12.5)	–	1 (5.9)
	Inpatient	–	1 (11.1)	1 (5.9)
	Leadership	1 (12.5)	–	1 (5.9)
	Primary Care	–	1 (11.1)	1 (5.9)
**Role**				
	Nursing	2 (25.0)	2 (22.2)	4 (23.5)
	Occupational Therapist	3 (37.5)	–	3 (17.6)
	Administrative	1 (12.5)	1 (11.1)	2 (11.8)
	Clinical/Counseling Psy.	–	2 (22.2)	2 (11.8)
	Psychiatrist	1 (12.5)	1 (11.1)	2 (11.8)
	Facilities Manager	1 (12.5)	–	1 (5.9)
	Pharmacist	–	1 (11.1)	1 (5.9)
	Support Worker	–	1 (11.1)	1 (5.9)
	Voluntary Services Manager	–	1 (11.1)	1 (5.9)

### Materials

All measures were self-completed and administered prior to the start of the first session of the course and at the end of the final session, unless stated otherwise. Six-month follow-up data was collected online with participants invited by email to complete measures *via* the Qualtrics survey platform.

### Parenting-Related Outcomes

Parental self-efficacy was assessed using TOPSE a 48-item scale developed to measure change in parenting self-efficacy across six domains: emotion and affection, play and enjoyment, empathy and understanding, control, discipline and boundary setting, pressures of parenting, self-acceptance, learning and knowledge (Kendall and Bloomfield, 2005). The scale is widely used to evaluate a range of parenting studies, has good reliability (Cronbach’s alpha = 0.94).

Parenting behavior was assessed using the 9-item Alabama Parenting Questionnaire-Short Form (APQ-SF) which assesses parenting practice in three domains: positive parenting, inconsistent discipline and poor supervision (Elgar et al., 2007). It has good internal consistency (Cronbach’s alpha = 0.68), test-retest reliability (0.84–0.90) and its sub-scales scales are sensitive to change.

The Strengths and Difficulties (SDQ) was administered as a child behavioral screen (Goodman et al., 2003). This 25-item screening tool measures parent reports of child behavior across five subscales: emotional problems, conduct problems, hyperactivity, peer relationship problems, prosocial behavior. It has been found to have good psychometric properties with a Cronbach’s alpha (0.84), and good test-retest reliability (0.62). It is sensitive to change and is widely used as screen for child psychopathology in community and clinical samples.

Ideographic goal: parents were asked to set a personal goal in attending the course. Parents provided a score of between one and 10 to describe their attainment toward the goal at the start and the end of the course.

### Parent Mental Health and Wellbeing Outcomes

Stress was measured using the stress sub-scale of the 21-item Depression, Anxiety and Stress Scale (DASS-21) (Lovibond and Lovibond, 1995). The seven item sub-scale has been used to measure stress in large populations and has been found to represent a distinct construct as well as a general dimension of negative affect (Henry and Crawford, 2005). The subscale possesses excellent internal consistency [Cronbach’s alpha = 0.90 (95% CI = 89–0.91)].

The Warwick Edinburgh Mental Wellbeing Scale (WEMWBS was) administered to assess wellbeing with regard to feeling and functional domains (Tennant et al., 2007). The WEMWBS is widely used in population-level and interventional research, has good sensitively to change and excellent and widely tested psychometric properties including Cronbach’s alpha score of 0.91, high test-retest reliability (0.83) and significant high correlation with measures of affect and life satisfaction (e.g., PANAS-PA *r* = 0.71, *p* < 0.01; SWLS *r* = 0.73, *p* < 0.01) (Stewart-Brown et al., 2009).

### Occupational Self-Efficacy

The 6-item Short-form Occupational Self-efficacy (OSE-SF) scale was administered to assess participants’ ability to manage the challenges of work and achieve their occupational goals (Rigotti et al., 2008). The scale has good convergent validity with significant partial correlations (controlled for age) with measures of job satisfaction (0.17–0.46, *p* < 0.05) and high levels of internal consistency (Cronbach’s alpha = 0.9) (Rigotti et al., 2008).

### Patient-as-Parent Practice

A secondary goal of the study was to explore whether the staff who took part increased in their use of parent-focused practice at work—i.e., thought about the parenting role of their clients. Parent-focused practice was measured using a 26-item scale developed by the research team for administration in a previous research project (Dunn et al., 2021). It comprised six items assessing the extent to which practitioners engage with the parenting role of patients in the service; nine items assessing practitioners’ attitudes and beliefs related to supporting “patient as parent” and 11 items assessing barriers to practice. These data will be reported elsewhere.

### Feasibility and Acceptability

Participant satisfaction and course acceptability was assessed using a nine-item scale administered at the end of the third course session. It comprised a series of brief questions [e.g., How enjoyable was the training? (very enjoyable/somewhat enjoyable/somewhat enjoyable/not enjoyable)] with an option to add further comment.

Acceptability of the course to participants’ managers was assessed using a brief online questionnaire comprising four yes/no questions relating to positive and negative effects of the course and an optional free text comment. This was administered to managers/team leads by email with the consent of participants. Two questions related to impact on “patient as parent practice” and are not reported here.

Project feasibility was evaluated using recruitment, sign-up and attrition data.

### Design and Procedure

The project was designed as a series of four parenting courses to be held at sites across East and West Sussex and Brighton and Hove. As discussed, two courses ended prematurely upon the introduction of COVID-19 restrictions. Of the two completed courses one took place in a community location and one took place on a clinical site. The courses comprised three 2.5-h sessions run over a four-week period, which included a one-week break for school holidays. Each course was led by two members of the study team: Group 1 was led by a clinical psychologist seconded to the trial from the from Child and Adolescent Mental Health Services (CD) and a research psychologist with expertise in the delivery of parenting interventions (AD); Group 2 was led by CD and a research and clinical psychologist with expertise in the design and delivery of specialist intervention for parents with anxiety (SCH). All facilitators have experience of working within the NHS and a good understanding of the Trust the intervention was located within.

Participants who had signed up for a course were contacted by the study team by email to remind of the location and duration of the worship and were invited to identify a goal in attending. The courses took place during paid work hours and participants were granted time out of their routine work to take part, and travel expenses were paid by the Trust. At the start of the first session consent was taken and baseline measures were administered.

The content of the course was developed by the research team with an awareness that each group could include participants holding a variety of roles and responsibilities and with varied levels of seniority. It was designed to offer participants an opportunity to share their experiences of parenting while working in a stressful mental health environment, and to foster skills and understanding around positive parenting and behavioral approaches. It incorporated content from a well-established intervention designed to promote positive parenting amongst parents who experience anxiety (Cartwright-Hatton et al., 2018) with additional material focused on work-life conflict in relation to operating in an emotionally and operationally demanding professional setting.

Further information about the course content is detailed in Table 2.

**TABLE 2:** Overview of the course content as delivered to participants. The bold line indicates the break point between sessions one, two and three.

Module	Module content
Introduction and ground rules	Including confidentiality. It was made clear that information divulged during sessions would not be relayed to managers.
Parenting identity	Rewards and challenges of being a parent
7 Confident thoughts	Core beliefs to promote child confidence
Parenting hotspots	Parenting behaviors which can undermine a child’s confidence
Attachment	Overview of core principles of attachment
Emotional coaching	Noticing and engaging with child’s emotions with empathy and active listening
Basic needs	Sleep, exercise, caffeine, diet
Play	Importance of play and specific methods of confidence-promoting play
Noticing, rewards and praise	Noticing child’s positive behavior and efforts with strategies for praise and rewards
Boundaries and limit setting	Importance of boundaries and “top tips” for using commands
Managing difficult behaviors	Emotion coaching approach to discipline

### Analyses

Descriptive characteristics for 17 participants were examined and outcomes scores were calculated. To maximize comparisons, data was analyzed using pairwise deletion.

Data were checked for normality of distribution using Kolmogorov-Smirnov tests with significance set at 0.05. Occupational self-efficacy (OSE-SF) and positive parenting (APQ-PP) data were non-normally distributed. Short-term intervention effects were calculated using pairwise *t*-tests with significance set at the 0.05 level. For variables which failed to meet the assumption of normality, Wilcoxon signed ranks were used. No comparisons were made using 6-month follow-up data due to the limited number of cases.

Data were analyzed in IBM SPSS 25 for Windows.

## Results

### Recruitment, Sign-Up and Attrition

Each of the four scheduled courses could accommodate 12 participants. Groups 1, 2 and 3 were fully booked (*n* = 36) with a combined waiting list of 33 participants. Ten participants signed up for Group 4.

Of the 46 participants who signed up to one of the four scheduled courses, 13 (28.26%) failed to attend the first session. Where a reason was provided, workload and illness were most commonly cited. Of the 17 parents who attended the first session of Group 1 (*n* = 8) or Group 2 (*n* = 9), 15 (88.24%) were present at the final session to complete post-intervention measures and six (35.29%) completed measures online at six-month follow-up.

### Outcome: Parenting Practice and Experience

On completion of the intervention, participants reported significantly higher levels of parental self-efficacy (*M* = 365.08, *SD* = 31.89) compared with baseline (*M* = 306.08, *SD* = 43.59), *t*(12) = 6.91, *p* < 0.05. The intervention was also associated with a significant reduction in use of inconsistent disciplinary strategies (*M* = 7.61, *SD* = 2.36 v *M* = 5.92, *SD* = 1.50), *t*(12) = 3.69, *p* < 0.005 and in child behavioral difficulties (*M* = 12.92, *SD* = 5.26 v *M* = 9.58, *SD* = 4.61), *t*(11) = 2.85, *p* < 0.005. At six-month follow-up, improvements in self-efficacy and reduction in inconsistent disciple remained elevated in comparison to baseline, however, due to the small number of cases, no formal statistical analysis was carried out. Participation was not associated with improvements in parental supervision or positive parenting activities.

### Outcome: Wellbeing, Stress, and Occupational Self-Efficacy

Participants reported a significant decrease in stress on completion of the intervention (*M* = 21.67, *SD* = 11.30 v *M* = 14.08, *SD* = 7.38), *t*(11) = 3.04, *p* < 0.05 with a smaller reduction maintained at 6-month follow up.

The courses were associated with an increase in wellbeing and occupational self-efficacy which remained above baseline at six-month follow up. These effects failed to reach significance at the *p* < 0.005 level.

### Outcome: Satisfaction

Of the seventeen participants who set a goal in attending the course, fifteen rated their progress toward it at the end of the final session. Participant goals related to engagement with their children (e.g., “to be more present”); managing behavior (e.g., “establishing consistent boundaries); their child’s anxiety (e.g., “new ways to support my child’s anxiety”); and to their own emotional state (e.g., “be less agitated”). There was a significant increase in participants’ reports of progress toward their goal after the course (*M* = 4.33, *SD* = 1.23 and *M* = 6.27, *SD* = 2.05), *t*(14) = 4.61, *p* < 0.005.

Thirteen participants completed course evaluation measures and all reported high levels of acceptability. In response to the following questions all participants selected the most positive response category: Would you recommend to colleagues? [yes = 100% (*n* = 13)]; How enjoyable was the training? [very enjoyable = 100% (*n* = 13)]; Did you find the format and timings of the courses acceptable? [yes = 100% (*n* = 13)]; Did you feel your manager supported your attendance? [yes = 100% (*n* = 12)].

Participants were given the option to add free text comments in relation to the statements above or to add further comment. Sixteen comments were received and have been organized into the following domains: 1) Satisfaction, eight participants commented positively on the facilitation, the benefit of sharing experiences with others or the utility of the content. E.g., “Kind supportive facilitators. Helpful amount of supporting material. Helped my wife and I think about parenting styles differences. Used resources with children.” No critical comments were received. 2) Scheduling, four participants expressed satisfaction with the format and duration of the course with two explicitly stating that it had been beneficial to participate during work hours. One recommended an earlier finish. 3) Managerial support, two participants stated they had not been supported to attend, with one choosing to attend on their day off and the second stating they were asked to miss a session but refused.

Managers/team leads reported that the courses had benefitted the wider team [yes = 71.4% (*n* = 5)], no = 28.6% (*n* = 2) and did not report any negative impact [no = 100% (*n* = 5)]. Four comments were received from managers which all stated the relevant participant had benefitted from attended, with two reporting that participants had shared strategies with other team members who were parents. No negative comments were received from managers.

## Discussion

The current project was designed to test the preliminary effectiveness of a workplace parenting intervention to deliver positive change for mental health workers, in terms of their parenting experience and practice, their mental health and wellbeing, and their self-efficacy in work. Given the pressures faced by the workforce in terms of capacity and resourcing, there was also a question of the feasibility of delivering this form of intervention and its acceptability to both participants and their managers. The study provides preliminary evidence that this approach is both effective and acceptable.

Staff who participated in the three-session courses reported increased parental self-efficacy and consistency in discipline. They also reported improvement in child psychological adjustment. The relationship between parental self-efficacy and positive parent and child outcomes is well-established (Albanese et al., 2019). Within the current context, it is arguable that the positive change reported for children was the result of parents developing skills to manage difficult behaviors and their increased confidence in their ability to perform their parenting role. Though there was reduction in the effects over time, after 6 months they remained elevated compared to baseline, indicating some longitudinal benefit of the training.

The courses were associated with a significant reduction in self-reported stress in participants, which declined from “moderate” to “normal” levels as categorized by the DASS. This is noteworthy given the program did not contain specific stress-management techniques, as was the case with Haslam’s work with the teaching workforce (Haslam et al., 2013). What is clear is that by ameliorating some of the challenges faced by parents of young children, the courses contributed to a reduction in the parents’ overall stress levels. This may relate specifically to improvements in the domestic sphere, for example a reduction in stress in response to reduced child behavioral difficulties. It could also be conceptualized as a positive “spill-over” as presented within the model of work-family conflict where improvements in either the home or work domain can have a protective effect in the other. The improvements in occupational self-efficacy may also reflect this inter-relationship, so that a greater belief in ability to function in the parenting role maps across to the professional domain.

The project also showed clear signs of feasibility and acceptability. The recruitment, participation and evaluation data all suggest that that delivery of this form of intervention is both practicable and desirable. Demand for places was high, with over-subscription at close to 100% and the attrition level was consistent with the mean rate identified in a meta-analysis of behavioral parent training programs (Chacko et al., 2016). The evaluation data suggests that the program met the needs of participants, which was further reinforced by the positive trend in response to participants’ ideographic goals.

The high levels of engagement with the program indicate there is a demand within the workforce for on-site support around parenting. However, any attempt to replicate the project must take into account some of the specific situational factors which may have contributed to its success. The Department of Health’s comprehensive review of workplace health interventions found that financial commitment from the organization, ease with which it can be taken up, intervention accessibility, and structures available to support participation are key to the success of an initiative (Brunton et al., 2016). The project under review contained four of these factors: it was funded by the Trust, participants were invited to participate during paid work time, with a range of course times and locations, and they were able to sign-up directly. Furthermore, the clear communication from the CEO of the Trust that the courses were developed with her support and a strategy of gaining buy-in from team leads further pulled the project in line with Bruton’s findings. In seeking to engage with staff who are parents, mental health trusts would be advised to replicate these approaches.

This was an exploratory project, designed to determine whether a brief parenting-focused course would be desirable and beneficial to staff working in the mental health sector. Even when taking into account the small sample, the results indicate the courses were of utility to participants, generating short-term effects in line with group-based parenting courses delivered in the community (Barlow and Coren, 2018). While the evidence of longer-terms effects should be interpreted cautiously given the low response rate, the positive trends are nonetheless noteworthy given that data was collected during the midst of a global pandemic. In reducing the stress levels of participants, the courses could offer a low-cost approach to scaffolding the health of the mental health workforce. This is particularly salient given the burden placed upon these workers as a result of the pandemic (Byrne et al., 2021). Now more than ever there is an imperative to deliver support which is acceptable and effective in promoting the mental health of the workers who have responsibility for looking after the mental health of the nation.

### Limitations

This was a feasibility study in which the Coronavirus pandemic led to a reduction in an already small sample. While the results are promising, they would need to be replicated with a larger sample before effectiveness can be determined with any certainty. Given the self-selecting nature of the sample, it is likely that participants were highly motivated which may have biased the results. However, outside of mandated parenting interventions, self-referral is a common mechanism in research and delivery of parenting interventions.

A further limitation of the study relates the demographic composition of sample. While research into parenting interventions largely involves female participants, the generalizability of the present study would benefit from reflecting the demographic make-up in terms of both gender and ethnicity of the Trust in which it as situated. Given the known high levels of stress in the mental health workforce there would additionally be benefit in capturing baseline mental health and disability data to enable disaggregated results.

### Next Steps

This study is a tentative first step toward the delivery of workplace-based parenting support for the mental health workforce and it would be valuably extended with a controlled trial with a sample large enough to unpick potential mediators. For example, does this form of intervention differentially benefit staff according to domains e.g., clinical and non-clinical? Sussex Partnership NHS Foundation Trust is one of the largest mental health trusts in the country and while it has reported above average levels of stress in its workforce, workers also report it to be more engaged in supporting their wellbeing compared with the national average (NHS England, 2021b). Given the variation in stress, work conditions, perceptions of managerial and organizational support at a trust level, there would be value in replicating the project in variety of trusts to better evaluate demand and acceptability.

The reduction in stress associated with participation indicates there is also utility in exploring whether the course has additional benefits in the professional domain, e.g., workplace-related stress or reduced absenteeism. Correspondingly, an economic evaluation is essential to determine value for money, particularly given the funding challenges faced by the sector.

## Data Availability Statement

The data that supports this findings of this study are openly available at Sussex Figshare (10.25377/sussex.19742320).

## Ethics Statement

The studies involving human participants were reviewed and approved by HRA and Health and Care Research Wales. The patients/participants provided their written informed consent to participate in this study.

## Author Contributions

AD conceptualized, designed and implemented the research, analyzed and interpreted data, and drafted and revised the manuscript. CD and SC-H conceptualized, designed and implemented the research and edited the manuscript. AT carried out project administration, supported data acquisition, and contributed to the revision of the manuscript. All authors contributed to the article and approved the submitted version.

## Conflict of Interest

The authors declare that the research was conducted in the absence of any commercial or financial relationships that could be construed as a potential conflict of interest.

## Publisher’s Note

All claims expressed in this article are solely those of the authors and do not necessarily represent those of their affiliated organizations, or those of the publisher, the editors and the reviewers. Any product that may be evaluated in this article, or claim that may be made by its manufacturer, is not guaranteed or endorsed by the publisher.
